# Uncertainties about the transmission routes of 2019 novel coronavirus

**DOI:** 10.1111/irv.12735

**Published:** 2020-03-04

**Authors:** Qingmei Han, Qingqing Lin, Zuowei Ni, Liangshun You

**Affiliations:** ^1^ The First Affiliated Hospital College of Medicine Zhejiang University Hangzhou Zhejiang China; ^2^ Department of Hematology The First Affiliated Hospital College of Medicine Zhejiang University Hangzhou Zhejiang China; ^3^ Malignant Lymphoma Diagnosis and Therapy Center The First Affiliated Hospital College of Medicine Zhejiang University Hangzhou Zhejiang China

The 2019 novel coronavirus (now named as SARS‐CoV‐2) caused an outbreak of SARS‐like illness in the late of December 2019. At present, the origin, susceptible population, and infection sources already have been clear.^1^, ^2^ However, the transmission routes, a key step to the epidemic control, have not yet been fully ascertained. Here, we focus on the potential transmission routes that have been investigated in the SARS‐CoV‐2 epidemic recently.

SARS‐CoV‐2, similar to SARS and MERS, is predominantly spread via respiratory tract with high infectivity. It is commonly recognized that droplet transmission is the main route. Spread by aerosol is suspected to be another important route of transmission but unestablished now. Epidemiological experts, as well as the WHO, consider more evidence is needed to confirm.^3^ Besides, there are other routes except respiratory transmission. The previous study indicated that different human coronaviruses, such as SARS‐CoV and MERS‐CoV, can maintain infectious for a different time on inanimate surfaces.^4^

Meanwhile, it was reported that SARS‐CoV‐2 was also founded on the surface of the door handles, cell phones, and other items in the residential sites of confirmed cases.^5^ Therefore, individuals will be probably infected if they touch the nose, mouth, or eyes after contacting the contaminated items.

Currently, the existing of live SARS‐CoV‐2 in the stool has been confirmed because researchers at two independent state key laboratories isolated this live virus from the stool specimens of cases with novel coronavirus‐infected pneumonia (NCIP).^6^ This phenomenon is coincident with that of SARS‐CoV as a result of ACE2 highly expressing in epithelia of the small intestine.^7^, ^8^ Furthermore, retrospecting the transmissibility of the SARS‐CoV in the 2002‐2003 pandemic, a shocking news reported that a major outbreak of SARS‐CoV involving 321 patients occurred in a high‐rise housing estate in Hong Kong.^9^ Sewage is considered to play a definite role in this transmission. In summary, fecal‐oral or fecal‐droplet mode of transmission may be one of the several routes and cannot be ignored.

Vertical transmission is another important mode of concern. A new retrospective study reviewed for nine pregnant women developing NCIP in late pregnancy at Zhongnan Hospital of Wuhan University suggests that no evidence is found for vertical transmission.^10^ Moreover, no SARS‐CoV‐2 was detected in breast milk, indicating that the virus may not be transmitted through breastfeeding neither.^10^ However, two newborns were confirmed in Wuhan women and children's healthcare center, one of whom was 30‐hour‐old, suggesting that the transmission via mothers to infants should not be ignored.^11^

Additionally, some researchers propose a hypothesis about the transmission through the ocular surface.^12^ Latest investigation reveals that although SARS‐CoV‐2 (1 of 67 cases) was tested positive in the conjunctival sac of NCIP patients, the hypothesis is not supported.^13^ Nevertheless, goggle is necessary to protect eyes for all medical workers.

Up to now, the outline of transmission routes of SARS‐CoV‐2 has not yet been drawn. More efforts must be made to get the full picture of transmission so that public health measures can be adopted timely by government to reduce further spread within China and beyond.

## CONFLICT OF INTEREST

The authors declare that they have no competing interests.

## FUNDING INFORMATION

The research was supported by National Natural Science Foundation of China (No. 81873451).
